# Atorvastatin lowers breast cancer risk by reversing an early tumorigenic signature

**DOI:** 10.1038/s41598-024-67706-2

**Published:** 2024-08-01

**Authors:** Mohamed Y. Foda, Mohamed L. Salem, Fadhl M. AlAkwaa, Omali Y. El-khawaga

**Affiliations:** 1 Biochemistry Division, Chemistry Department, Faculty of Science, Mansoura University, Mansoura, 35516 Egypt; 2 Immunology and Biotechnology Unit, Department of Zoology, Faculty of Science, and Center of Excellence in Cancer Research, Tanta University, Tanta, Egypt; 3 Division of Nephrology, Department of Internal Medicine, University of Michigan, Ann Arbor, MI USA

**Keywords:** Biochemistry, Cancer

## Abstract

Breast cancer remains a significant health challenge with complex molecular mechanisms. While many studies have explored genetic markers in breast carcinogenesis, few have studied the potential impact of pharmacological interventions such as Atorvastatin on its genetic landscape. This study aimed to elucidate the molecular distinctions between normal and tumor-adjacent tissues in breast cancer and to investigate the potential protective role of atorvastatin, primarily known for its lipid-lowering effects, against breast cancer. Searching the Gene Expression Omnibus database identified two datasets, GSE9574 and GSE20437, comparing normal breast tissues with tumor-adjacent samples, which were merged, and one dataset, GSE63427, comparing paired pre- and post-treated patients with atorvastatin. Post-ComBat application showed merged datasets' consistency, revealing 116 DEGs between normal and tumor-adjacent tissues. Although initial GSE63427 data analysis suggested a minimal impact of atorvastatin, 105 DEGs post-treatment were discovered. Thirteen genes emerged as key players, both affected by Atorvastatin and dysregulated in tumor-adjacent tissues. Pathway analysis spotlighted the significance of these genes in processes like inflammation, oxidative stress, apoptosis, and cell cycle control. Moreover, there was a noticeable interaction between these genes and the immunological microenvironment in tumor-adjacent tissues, with Atorvastatin potentially altering the suppressive immune landscape to favor anti-tumor immunity. Survival analysis further highlighted the prognostic potential of the 13-gene panel, with 12 genes associated with improved survival outcomes. The 13-gene signature offers promising insights into breast cancer's molecular mechanisms and atorvastatin's potential therapeutic role. The preliminary findings advocate for an in-depth exploration of atorvastatin's impact on.

## Introduction

Breast cancer remains the most prevalent malignancy and major cause of death in females worldwide^1^. Breast cancer patient prognosis has been greatly improved with advances in cancer therapy^2–4^. Breast cancer is a heterogeneous disease influenced by epigenetic, genetic, and environmental factors^5^. Recent studies on the mechanisms underlying breast cancer have yielded significant advancements, revealing the initiation and progression of breast cancer^6,7^. However, the carcinogenic pathways and novel pathogenic genes still need further investigation. The journey from normal to malignant tissue embodies a convoluted, multi-step process, characterized by an array of cellular and molecular alterations^8,9^. One critical aspect of this exploration is the identification and understanding of differentially expressed genes (DEGs) in breast tissues, specifically distinguishing between normal and tumor-adjacent samples. These DEGs can provide invaluable insights into the pivotal changes that push normal breast tissues towards a tumorigenic state, offering potential targets for therapeutic intervention and prognosis^10,11^. In addition to the intrinsic genetic factors, the tumor microenvironment, characterized by a dynamic interaction between cancer cells and host immune cells, plays a cardinal role in tumor progression, metastasis, and therapeutic resistance. The infiltration patterns of various immune cell types within the tumor’s vicinity can dictate the tumor's aggressive behavior and its response to therapies. A thorough understanding and strategic targeting of these early alterations stands as a promising avenue in our combat against breast cancer.

A study by Kang et al. focused on transcriptional differences in histologically normal cancer-adjacent breast tissues. They identified a multi-gene signature capable of distinguishing normal breast samples expressing either an active or inactive transcriptome phenotype. The active phenotype was associated with increased expression of genes linked to tumorigenesis^12^. Roman-Perez et al. examined gene expression in the extra tumoral microenvironment and its clinical outcome in breast cancer patients. They found that some cancer patients have gene expression patterns in their adjacent non-neoplastic tissue that are similar to invasive breast cancer signatures. These signatures may predict the progression of early premalignant lesions^13^. Tripathi et al. employed oligonucleotide microarrays to analyze RNA from micro dissected tissues of two distinct groups: cancer normal epithelium (CNE) and reduction mammoplasty (RM). They found that global gene expression abnormalities exist in CNE of breast cancer patients and are also present in early cancers. Thus, cancer-related pathways may be perturbed in normal epithelium^10,14^. A similar study by Graham et al. using expression microarrays found a distinct signature distinguishes the normal epithelium of breast cancer cases, including both estrogen receptor positive (ER+) and ER− cancers, from that of controls (reduction mammoplasty samples), and this profile can be discerned in prophylactic mastectomy (PM). This suggests that the histologically normal (HN) profile is not an effect of the tumor, but instead may be a marker of increased breast cancer risk, or a reflection of breast cancer's earliest gene expression changes^11^.

The protective effects of drugs against diseases by reversing disease signatures is an evolving field of research, primarily explored through the lens of transcriptomics, which involves the study of RNA transcripts to understand gene expression changes associated with diseases. A study highlighted the concept of Transcriptomic Signature Reversion (TSR) for drug repositioning and repurposing in various cancer types. The underlying assumption of TSR is that drugs capable of reverting disease-induced gene expression changes back to healthy levels are likely to be effective against the disease^15^.

Statins, particularly atorvastatin, commonly prescribed to manage hypercholesterolemia through inhibit HMG-CoA reductase in the mevalonate pathway, exhibit a spectrum of effects extending beyond their primary cardiovascular applications^16–18^. Atorvastatin has emerged as intriguing candidates in cancer therapeutics due to preliminary evidence suggesting its role in modulating the breast cancer transcriptome and potentially reversing early cancer driver signatures^19,20^. While its primary role in reducing cardiovascular risk by curtailing LDL cholesterol is well-established, burgeoning evidence suggests atorvastatin's potential anti-cancer properties, particularly in breast cancer^21^.

The sustained use of lipophilic statins for 1 year or more before a breast cancer diagnosis significantly reduced the proportion of ER/PR-negative tumors in comparison to no statin use or use for less than 1year (OR 0.63, 95% CI 0.43–0.92; *p* = 0.02)^22^. Also, this reduced the proportion of late-stage breast cancer with hazard ratio (HR 0.80, 95% CI 0.64–0.98, *p* = 0.035), especially among estrogen receptor ER + tumors (HR 0.72, 95% CI 0.56–0.93, *p* = 0.012) in comparison to no statin use. Furthermore, breast cancer mortality was marginally lower in statin users compared with nonusers (HR 0.59, 95% CI 0.32–1.06; *p* = 0.075)^23^. A genome-wide associations study (GWAS) of the Single-nucleotide polymorphisms (SNPs) in HMGCR found that genetically proxied inhibition of HMG-CoA reductase was significantly associated with a reduced risk of breast cancer (OR 0.84; 95% CI 0.74–0.95; *p* = 0.005), thus variants leading to the inhibition of HMG-CoA reductase lowered the probability to develop breast cancer. Further analysis revealed that the reduced risk associated with genetically proxied inhibition of HMG-CoA reductase was primarily observed for ER+ breast cancer (OR 0.82, *p* = 0.008)^24^. The study revealed a significant correlation between the use of statins and a reduced risk of breast cancer. Among the cohort of 40,421 women studied, 4771 were on statins, constituting 11.8% of the total participants, breast cancer was observed in 1.38% of the participants. Intriguingly, the data indicated that statin users were significantly less likely to develop breast cancer (OR 0.49; 95% CI 0.38–0.62, *p* =  < 0.0001)^25^. The utilization of statins, particularly lipophilic types like atorvastatin, demonstrates a notable association with the risk reduction of breast cancer. Specifically, the Potential Outcome Mean (POM) estimates, which represent the predicted risks of breast cancer, were lower for both lipophilic (0.0038; 95% CI: 0.002, 0.0056) and hydrophilic statin users (0.0051; 95% CI: 0.0008, 0.0095) in comparison to non-statin users (0.0072; 95% CI: 0.0055, 0.0089). Moreover, the Average Treatment Effect (ATE), which refers to the risk difference, was found to be significantly negative for lipophilic statins (-0.0034; 95% CI: -0.0059, -0.0009), indicating a reduced risk of breast cancer associated with their use.

The risk difference for hydrophilic statins, while negative, was not statistically significant (-0.0021; 95% CI: -0.0067, 0.0026). These results also provide an insight into the number needed to treat (NNT), suggesting that approximately 3.4 cases of breast cancer could be prevented per 1000 subjects treated with lipophilic statins like atorvastatin. However, the prevention rate for hydrophilic statins, though calculated at 2.1 cases per 1000, was not found to be statistically significant^26^. Pooled analysis of studies examining pre-diagnosis statin use and breast cancer survival revealed a negative association with overall survival (HR 0.68, 95% CI 0.54–0.84) and disease-specific survival (HR 0.72, 95% CI 0.53–0.99). However, there was high heterogeneity across studies. Regarding post-diagnosis statin use, while no statistically significant association was found for overall survival (HR 0.71, 95% CI 0.48–1.07), a significant negative association was observed for disease-specific survival (HR 0.65, 95% CI 0.43–0.98)^27^.

We hypothesize that statins may possess the potential to reverse or impede the early oncogenic signatures in breast tissue, thereby obstructing its progression to malignancy. This investigation is predicated on unraveling the protective mechanism by Atorvastatin against breast cancer onset, with a focal point on its influence over the early oncogenic alterations within normal breast tissue. The methodology of this investigation is structured to examine the interplay between Atorvastatin treatment and early oncogenic signatures within breast tissue. Initially, the analysis will focus on identifying commonly regulated genes in paired Atorvastatin pre- and post-treated patients’ samples and tumor-adjacent samples compared to a normal reduction mammoplasty samples to understand the molecular dialogue entailed. Following this, a differential immune cell infiltration analysis using immune deconvolution techniques will be conducted in both cohorts to explore the immunomodulatory effects of Atorvastatin. The significance of the overlap in gene regulation between these groups will be calculated, with a subsequent functional enrichment analysis on identified genes to elucidate their roles in cellular processes and pathways. Lastly, the expression levels and prognostic significance of these genes will be examined to assess their potential as predictive markers for breast cancer incidence and progression. Through this integrative approach, the study aims to delineate the molecular and immunological mechanisms by which Atorvastatin may intervene in the early oncogenic transitions within breast tissue, hoping to contribute a novel preventive paradigm against breast cancer.

## Materials and methods

### Data retrieval

Our data search and retrieval process were carried out using the Gene Expression Omnibus (GEO; https://www.ncbi.nlm.nih.gov/geo/) database^28^, a resource that provides access to a variety of microarray, next-generation sequencing, and other forms of high-throughput functional genomics data. Our search strategy focused on datasets related to "breast cancer," specifically including the terms "tumor adjacent" and "reduction mammoplasty" in the search query using the advanced search. The inclusion criteria were human samples with at least 20 samples and the presence of tumor-adjacent or reduction mammoplasty samples. The exclusion criteria were non-human studies and studies with fewer than 20 samples. We identified two datasets, GSE9574 and GSE20437, both of which were generated using the GPL96 Affymetrix Human Genome U133A Array platform. The GSE9574 dataset comprises 15 samples from reduction mammoplasty procedures and 14 samples from tumor-adjacent tissues. The GSE20437 dataset includes 18 samples each from reduction mammoplasty procedures and tumor-adjacent tissues.

To further investigate the role of Atorvastatin in breast cancer prevention, we conducted a separate search with the keywords "Atorvastatin" and "breast cancer" using advanced search. This search had similar criteria but specifically required studies with atorvastatin treatment paired samples. This led to the identification of the GSE63427 dataset, which was generated using the Illumina HumanHT-12 V4.0 expression beadchip. However, upon inspection of the GSE63427 dataset, we noted ambiguity in the sample annotation. Thus, six samples were excluded from further analysis due to this uncertainty. The raw CEL files for GSE9574 and GSE20437 were directly downloaded from the GEO database, while the GSE63427 expression matrix was obtained using the GEOquery package in R^29^.

### Microarray data preprocessing and identification of DEGs

Microarray data corresponding to datasets GSE9574 and GSE20437 were independently preprocessed. Raw Affymetrix CEL files were imported using the ReadAffy function implemented in the Affy package in R. Quality control (QC) procedures were carried out in two distinct rounds using the ArrayQualityMetrics package in R. In the initial phase, arrays failing at least two of the six utilized QC metrics were excluded prior to normalization. These metrics comprised outlier detection strategies via Distances between arrays, Boxplots, Relative Log Expression (RLE), Normalized Unscaled Standard Error (NUSE), MA plots, band Spatial distribution of M. Upon successful passage through the first round of QC, the remaining samples underwent preprocessing and normalization via the Robust Multi-array Average (RMA) method. Post-normalization, a second round of QC was conducted. Arrays that failed any of the following three metrics, namely, Distances between arrays, Boxplots, and MA plots were subsequently omitted from further analyses.

The processed datasets were subjected to exploratory analysis via the construction of boxplots, densities, and Multidimensional Scaling (MDS) plots. Following this, the two datasets were merged, with the potential batch effects adjusted using the ComBat function from the sva package in R^30^.

Principal Component Analysis (PCA) was carried out on the merged datasets based on the top variable 500 probes to assess the patch effect and visualized using the ggplot2 packages in R. Probe level data was annotated using gene symbols with the assistance of the hgu133a.db package in R. Genes mapped to multiple probes were aggregated by calculating their mean values. Differential gene expression analysis was conducted between two tissue types, namely Normal Breast and Tumor Adjacent for the merged and the GSE63427 datasets, using the limma package in R^31^. The differentially expressed genes (DEGs) were screened based on the following criteria: a log2 fold change (FC) > =|0.585| and a false discovery rate (FDR) < 0.05. DEGs were visualized via volcano plots using the EnhancedVolcano package in R. Moreover, heatmaps were constructed to showcase the top 100 significant DEGs using the ComplexHeatmap R package. We used venny to calculate the common genes between platforms and the commonly regulated genes in both datasets. We treated these genes as early oncogenic target signature.

### Construction of PPI network and PCA of the 13 genes based on GETx and TCGA-BRCA samples

A protein–protein interaction (PPI) network was constructed by uploading the genes to the Search Tool for the Retrieval of Interacting Genes/Proteins database (STRING v.12.0; https://string-db.org/)^32^. The settings for building the PPI network were established in accordance with the “Homo sapiens” model, and the confidence of the interaction between the targets was set at 0.4. The network nodes represented proteins, and the edges reflected the protein–protein interactions.

PCA was performed using the web-based tool, Gene Expression Profiling Interactive Analysis 2 (GEPIA2; http://gepia2.cancer-pku.cn/)^33^. GEPIA2 is a robust online resource that allows the user to perform PCA using a selected set of genes across pre-analyzed data from the Genotype-Tissue Expression (GTEx)^34^ and The Cancer Genome Atlas Pan-Cancer Analysis (TCGA)^35^ projects. To investigate whether the selected genes could separate normal tissue from tumor and tumor-adjacent samples, the 13 genes were inputted into the Dimensionality reduction module of GEPIA2. The resulting PCA was assessed to determine whether distinct clusters could be identified that represented the GTEx normal samples, the TCGA breast cancer (BRCA) tumor samples, and the TCGA-BRCA tumor-adjacent samples. Further analysis was conducted to explore if the 13 genes could differentiate between the normal samples, whether they originated from GTEx or as tumor-adjacent samples from TCGA-BRCA, and the TCGA-BRCA tumor samples.

### Functional and gene set enrichment analysis

Functional enrichment analysis was performed using the Enrichr web tool (https://maayanlab.cloud/Enrichr/)^36^. Enrichr is a comprehensive gene set enrichment analysis web server. The commonly regulated 13 genes were uploaded to the server for analysis. Enrichr offers several gene set libraries to choose from. In this study, we focused on the following databases: the Molecular Signatures Database (MSigDB)^37^, the Kyoto Encyclopedia of Genes and Genomes (KEGG) pathways^38^, Gene Ontology (GO)^39^ Biological Processes and Molecular Function. After inputting our gene sets, the enrichment analysis was performed, with the results visualized as barplots and interpreted accordingly. Enrichr calculates the enrichment of each gene set using a combination of Fisher exact test and correction for multiple hypotheses testing. Enriched terms with adjusted *p* value less than 0.05 were considered significantly enriched. The combined score, which is a combination of the *p* value and z-score, was used to rank the gene sets.

To assess pathway-level enrichment the differentially expressed genes in the altered expression group, we used blitzGSEA^40^, a Python package for the computation of Gene Set Enrichment Analysis (GSEA) (https://github.com/MaayanLab/blitzgsea). Genes were ranked based on the log2FC and used as input. The top significantly enriched or depleted terms were plotted.

### The evaluation of immune cells in merged and GSE63427 datasets

CIBERSORT^41,42^, a deconvolution algorithm based on normalized gene expression profiles, can quantify the immune cell composition and has greatly expanded the potential of the genomic database. For our analysis, we used the LM22 signature matrix, which includes 22 immune cell types, allowing a comprehensive evaluation of the immune cell composition. We used the expression data of the genes commonly found in the GPL96 Affymetrix Human Genome U133A Array and the Illumina Human HT-12 V4.0 expression beadchip as input to CIBERSORT. This approach was taken to avoid statistical bias. Each updated matrix was uploaded to the CIBERSORTx website (https://cibersortx.stanford.edu/). We imputed the absolute cell fractions, performed batch effect correction, and ran 1000 permutations for each iteration.

For the merged dataset, we applied the t-test for normally distributed cells and the Mann–Whitney U test for cells not normally distributed. For the GSE63427 samples, we performed paired t-tests for the normally distributed cells and used the Wilcoxon signed-rank test for cells not normally distributed. The significantly differentially infiltrated immune phenotypes were visualized using heat maps and box plots. Additionally, we explored the correlation between the expression of the 13 genes and the immune cells in tumor-adjacent and normal breast samples. These correlations were visualized using the corrplot package.

### Correlations with gene expression alterations of the 13 genes

This study utilized The cBio Cancer Genomics Portal (cBioPortal; https://www.cbioportal.org/)^43^, a comprehensive web resource providing visualization, analysis and download of large-scale cancer genomics data sets, to investigate the expression pattern of 13 genes within the TCGA-BRCA (The Cancer Genome Atlas—Breast Invasive Carcinoma) cohort. We utilized the comparison module of cBioPortal to classify the TCGA-BRCA samples into two distinct groups based on their z-scores from the scaled expression data. Samples with a z-score greater than 2 or less than -2 were categorized as 'altered', indicating significant deviation from the norm, while those within these thresholds were classified as 'unaltered'. This categorization forms the basis for our comparative analysis between these groups. Subsequently, an intensive analysis was conducted on the key clinical attributes associated with these gene alterations. Further exploration was carried out on the genomic alterations themselves in both groups, allowing for a comprehensive understanding of the genetic landscapes. Moreover, differentially expressed genes between the altered and unaltered groups were identified. This was crucial to understand the functional impact of the alterations, thereby shedding light on the potential pathogenic or therapeutic implications these changes could have in breast invasive carcinoma. Furthermore, we utilized the The University of ALabama at Birmingham CANcer data analysis Portal (UALCAN; https://ualcan.path.uab.edu/) database^44^, a comprehensive resource for studying cancer OMICS data. In particular, UALCAN was used to explore the expression of the 13 genes of interest between TP53 mutant and non-mutant samples. This approach provided a deeper understanding of the role these genes play in TP53 mutation status.

### Survival analysis

We used the bc-GenExminer database to assess the prognostic value of the 13 gene expression. Using the prognostic module^45^, all the RNA-seq data were used to analyze the correlation between the gene panel expression and both overall survival (OS) and disease-free survival (DFS). Targeted analysis was performed to evaluate the overall prognostic value.

## Results

### Identification of DEGs

**Figure 1:**
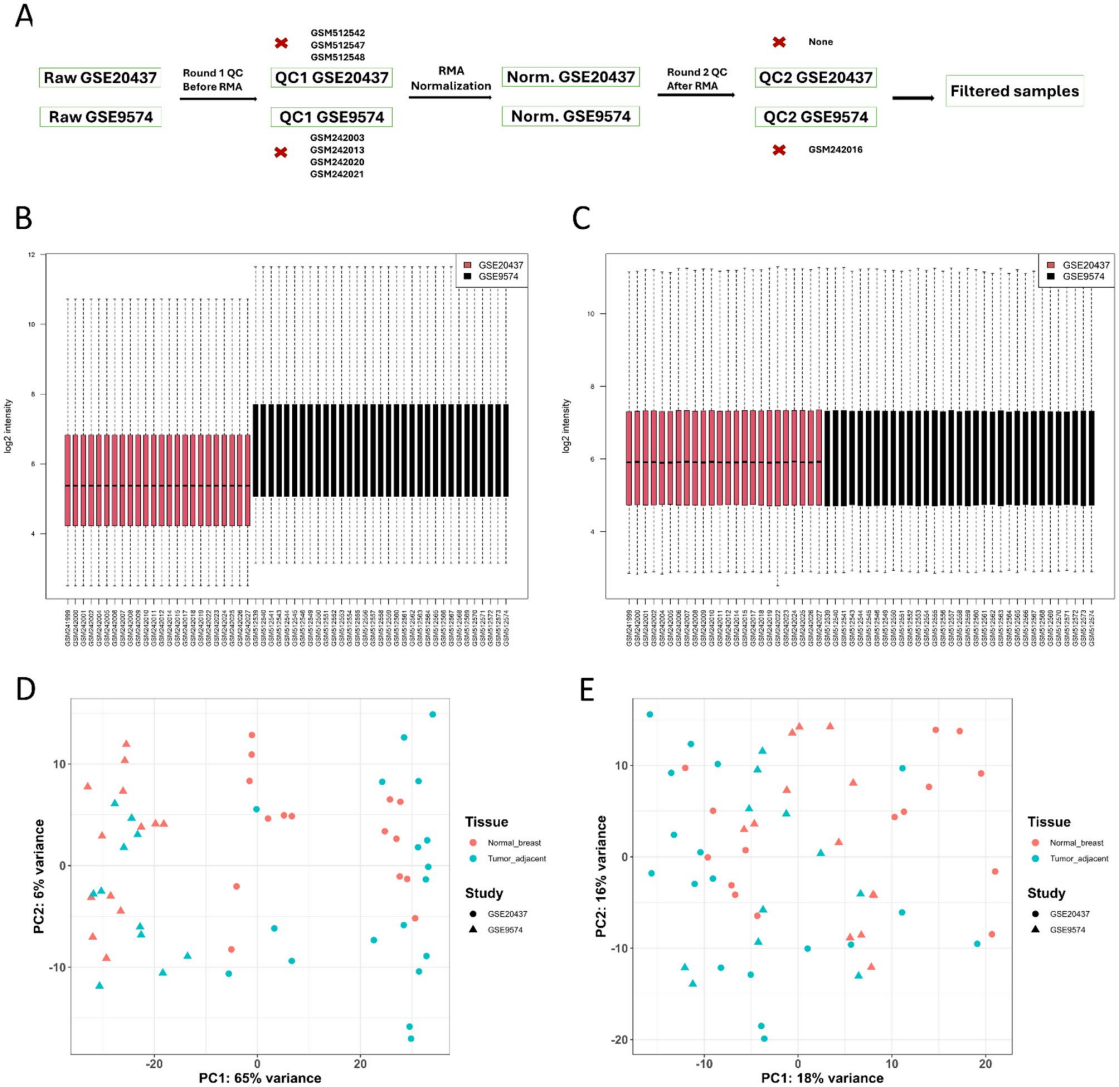
QC and Preprocessing and merging the GSE20437 and GSE9574 datasets. (**A**) Steps of Quality control steps and preprocessing. Red x indicates the excluded samples in each step. (**B**) Boxplots of the merged data sets before applying Combat, a clear batch effect between the datasets. (**C**) Boxplot of the merged dataset after applying Combat and removing batch effect, samples have similar intensity disrtibution thus comparable. (**D**) PCA of the merged datasets before applying Combat based on top 500 variable genes. (**E**) PCA of the merged datasets after applying Combat and removing batch effect based on the top 500 variable genes.

Following the rigorous quality control procedures and data preprocessing (Fig. 1A), a total of 4 samples from the GSE9574 dataset and 3 from the GSE20437 dataset failed the first round of QC (Table S1, Supplementary Files 1, 2). After RMA normalization, an additional sample from GSE9574 failed to meet the QC metrics in the second round (Table S2, Supplementary Files 3, 4). Consequently, a total of 57 out of the initial 65 samples were considered for further analysis. The potential batch effects between the two datasets were effectively adjusted post ComBat application. (Fig. 1B,C) where a unified distribution of both studies was observed post-ComBat application.

Principal Component Analysis (PCA) was employed to evaluate the batch effect before and after normalization. The PCA plot indicated a decreased impact of batch effects after ComBat adjustment. Importantly, no clear separation between the datasets was observed, further indicating the reduction of batch effects. Given the nature of the samples (normal breast tissue and tumor adjacent breast tissue), this was an anticipated outcome (Fig. 1D,E).

**Figure 2:**
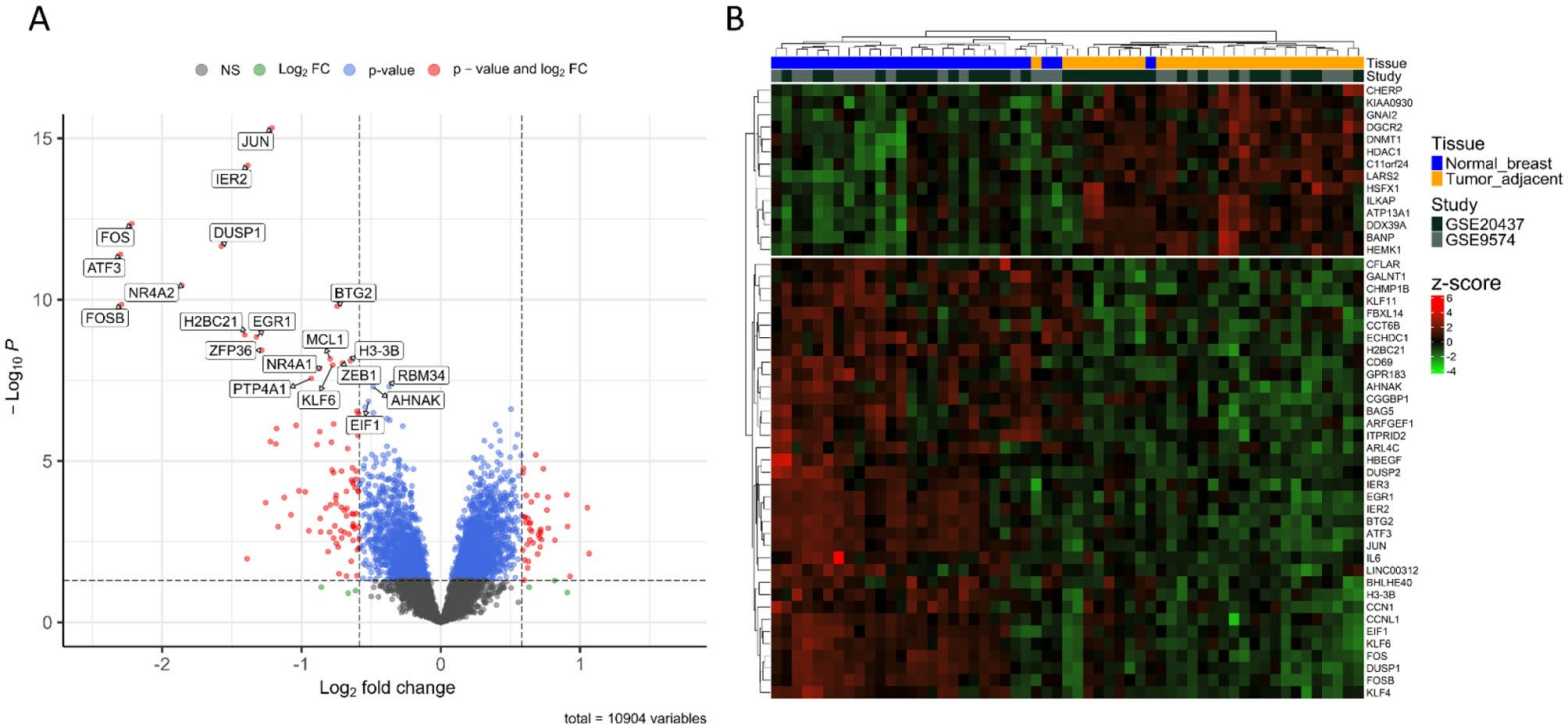
Diffrentially expression analysis of normal and tumor adjacent samples. (**A**) Volcano plot of the DEGs between normal and tumor adjacent samples. (**B**) Heatmap of the top 50 significant diffrentially expressed genes between normal and tumor adjacent samples.

Using the limma package in R and according to the applied criteria, a total of 116 genes were identified as differentially expressed between normal breast and tumor adjacent tissues. Of these DEGs, 33 genes were upregulated, while 83 genes were downregulated (log2 FC > =|0.585| , FDR < 0.05). The distribution and statistical significance of these DEGs were visualized through a volcano plot (Fig. 2A). To further emphasize the differential gene expression, a heatmap was constructed based on the top 50 DEGs (Fig. 2B). This heatmap demonstrated a clear clustering between the samples, thereby underlining the robustness of the gene expression changes observed.

These findings illuminate the molecular differences between normal and tumor-adjacent breast tissues. The distinct up- and downregulation of certain genes suggest intricate molecular mechanisms at play in the transition from normal to tumor-adjacent tissue. The identified DEGs offer a promising basis for further investigations into the mechanisms underlying breast carcinogenesis and potential therapeutic targets.

**Figure 3:**
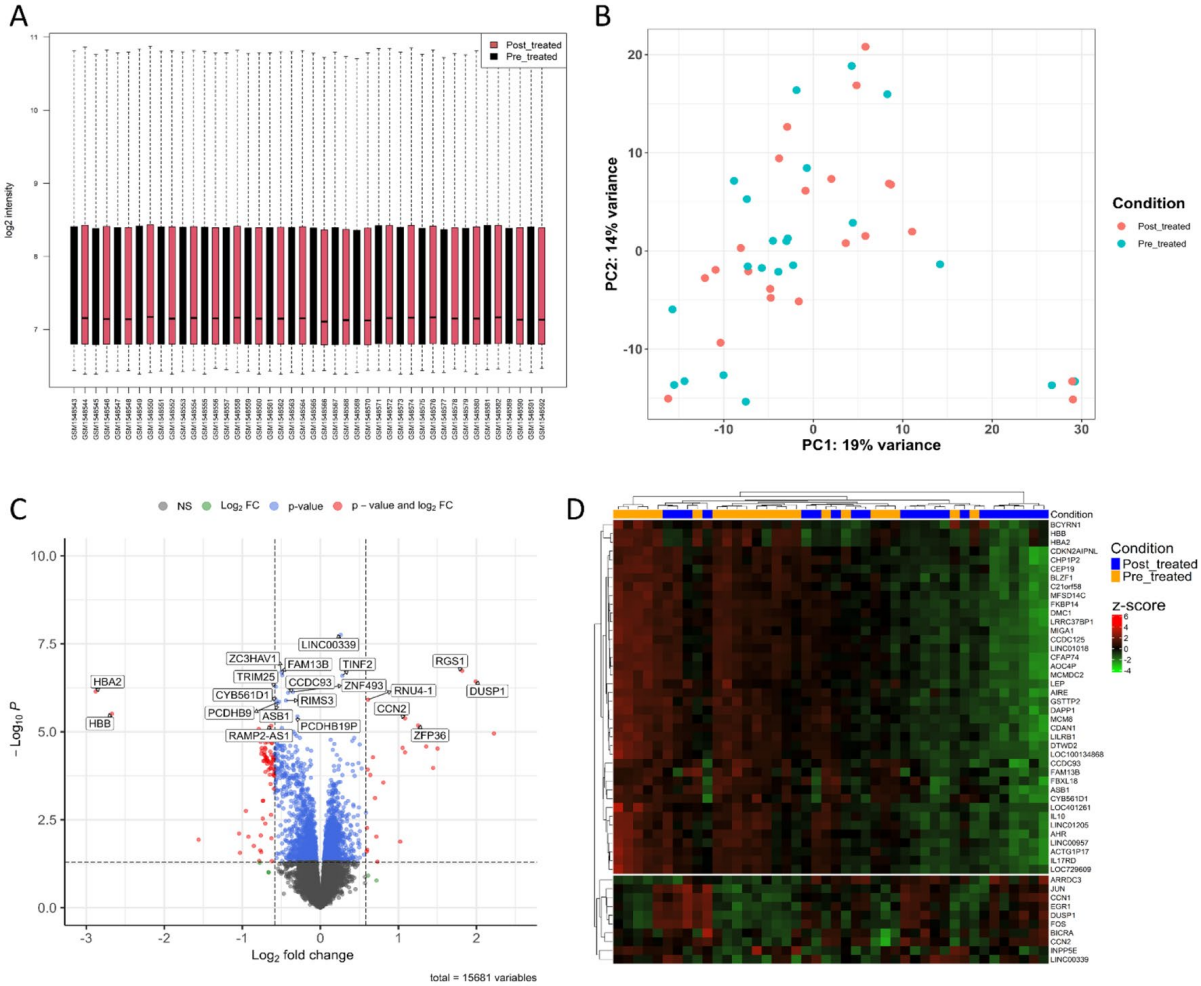
Preprocessing and differential expression analysis of pre and post-treated samples. (**A**) Intensity distribution boxplot of the pre and post treated samples. Data were normalized. (**B**) PCA of the pre and post-treated samples based on top 500 variable genes. (**C**) Volcano plot of the DEGs between pre and post-treated samples. (**D**) Heatmap of the top 50 significant diffrentially expressed genes between pre and post-treated samples.

In order to study the impact of the drug in question “atorvastatin” on breast cancer, we utilized patients’ samples from the GSE63427 data downloaded from GEO. Of these, 6 samples were excluded as they were suspected to be mislabeled. The assessment of expression intensities via boxplot indicated comparable distribution profiles between the samples, obviating no need for normalization (Fig. 3A). A principal component analysis (PCA) was conducted to examine the overall effect of Atorvastatin treatment. The results suggested a lack of clear separation between the pre- and post-treated samples (Fig. 3B). This finding indicates that the impact of Atorvastatin at the provided dose over a two-week period appears to be minor.

Following this, a paired t-test was carried out with the aid of the limma package to further investigate the differential gene expression between the pre- and post-treatment samples. A total of 105 DEGs were identified; of these, 58 genes exhibited downregulation post-treatment, while 16 genes were upregulated according to the applied criteria (Fig. 3C). To facilitate the visualization of DEGs, a volcano plot was generated. This graphical representation provided a comprehensive overview of the significant alterations in gene expression caused by the Atorvastatin treatment. In addition, a heatmap was constructed based on the top 50 most significantly altered DEGs (Fig. 3D). Interestingly, this heatmap demonstrated a degree of clustering among samples, thereby providing a snapshot of the relationships and patterns inherent in the data.

### Atorvastatin responsive signature is downregulated in tumor-adjacent tissue

**Figure 4:**
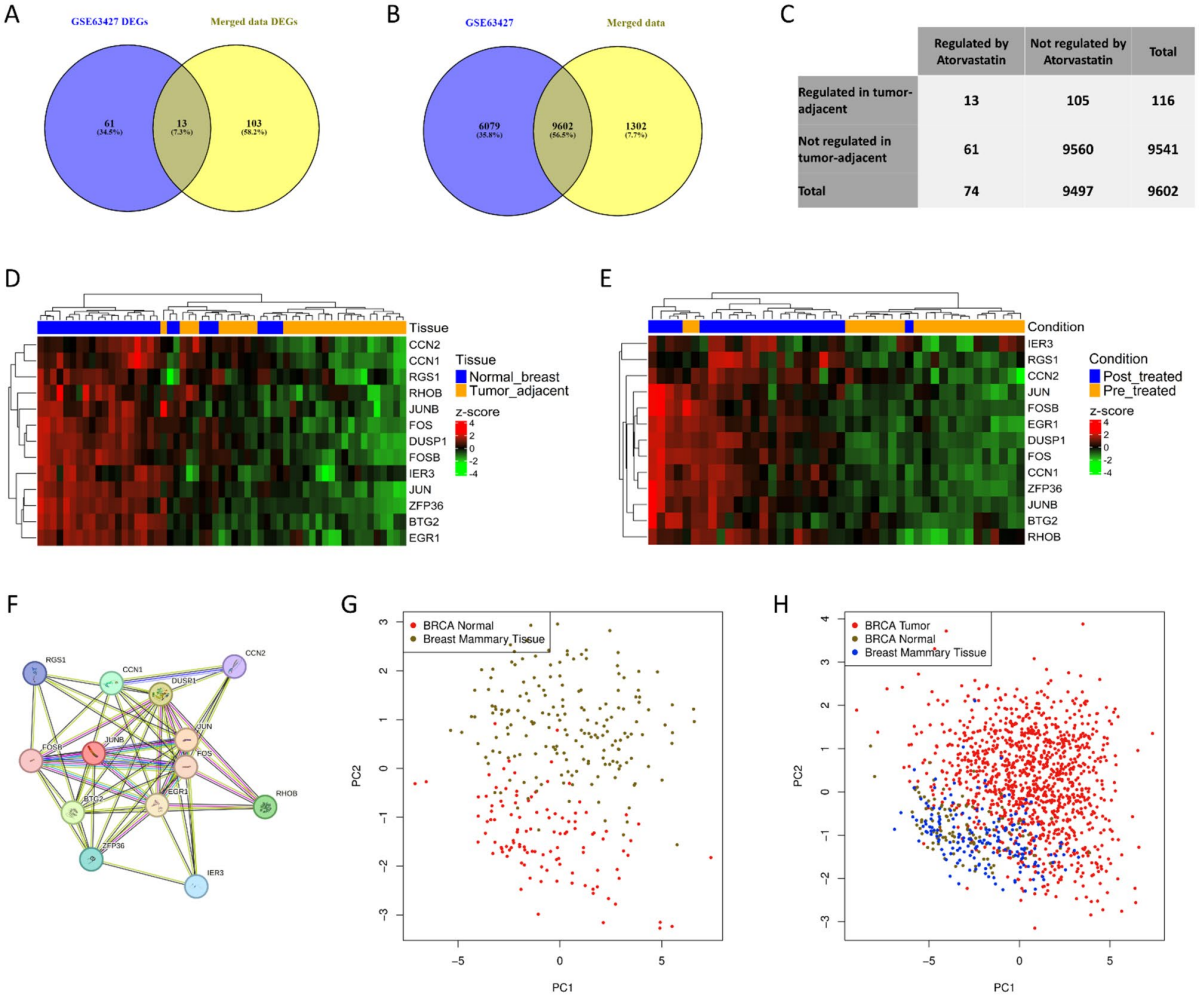
Analysis of the commonly regulated 13 genes between the merged and GSE63427 datasets. (**A**) Venn diagram of the genes commonly regulated in merged and GSE63427 datasets. (**B**) Venn diagram of the common genes in GPL96 Affymetrix Human Genome U133A Array and Illumina HumanHT-12 V4.0 platforms. (**C**) Contingency matrix of the hypergeometric test for the 13 commonly regulated genes. (**D**) Heatmap of the 13 commonly regulated genes in the merged dataset. Normal and tumor adjacent samples clustered based on the 13 genes expression. (**E**) Heatmap of the 13 commonly regulated genes in the GSE63427 dataset. Pre and post treated samples clustered based on the 13 genes expression. (**F**) PPI netweok of the 13 commonly regulated genes from STRING database. Genes were highly connected. (**G**) PCA of the 13 genes in normal GTEx (green) and tumor adjacent TCGA-BRCA (red) samples using GEPIA2 webtool. The genes separated normal and tumor adjacent samples. (**H**) PCA of the 13 genes in GTEx (green), tumor adjacent TCGA-BRCA (red) and breast cancer (blue) samples using GEPIA2 webtool. The genes separated normal samples- either GTEx of TCGA-BRCA normal samples- and breast cancer samples.

The primary objective of our investigation was to assess the potential protective effect of Atorvastatin against the incidence of breast cancer. Our hypothesis was that Atorvastatin could potentially reverse an early cancer driver signature, thereby reducing the likelihood of disease onset. To explore this hypothesis, we conducted an intersection of the genes regulated by Atorvastatin in breast cancer with those that were dysregulated in breast tumor-adjacent tissue. This analysis yielded 13 genes that were commonly influenced (Fig. 4A).

To ascertain whether this overlap occurred by coincidence or represented a biologically significant connection, we executed a hypergeometric test. The set of background genes was considered to include those shared across both the atorvastatin-regulated and tumor-adjacent dysregulated platforms. This intersection resulted in a common set of 9602 genes (Fig. 4B). The contingency table of the hypergeometric test is shown in Fig. 4C. The results of the hypergeometric test generated a *p* value of 1.57e-13. This significantly low *p* value suggests a statistically meaningful enrichment rather than a random coincidence. Therefore, the 13 genes commonly regulated by Atorvastatin and dysregulated in breast tumor-adjacent tissue may not be just due to chance, suggesting that Atorvastatin indeed has potential implications in the context of early cancer driver signature. In order to delve deeper into the function and interplay of the identified 13-gene signature, we visualized their expression patterns in normal and tumor-adjacent tissues, as well as pre- and post- Atorvastatin treatment, using heatmaps. This analysis revealed an opposite pattern of expression in these comparisons, indicating a possible reversal of the dysregulated gene signature by Atorvastatin treatment (Fig. 4D,E).

The protein–protein interaction (PPI) network analysis of the 13 identified genes, was generated using the STRING database (Fig. 4F). The network was densely connected, comprising 13 nodes and 54 edges and enrichment of *p* value less than 1.0e-16, which suggests a strong interconnectedness among the encoded proteins. An average node degree of 8.31 and average local clustering coefficient of 0.844 was observed, indicative of the high connectivity in the network and high tendency to form closely knit clusters or groups. This dense interconnectivity suggests that these proteins may not act independently but rather exhibit coordinated functionality. This could imply a shared or cooperative functionality among them. Based on this, we decided to keep all the genes for downstream analysis.

Building upon these results, we further deepened our analysis through the application of the GEPIA2 tool for gene expression profiling. This was specifically done to validate whether the gene signature could effectively distinguish normal tissue (from GTEx) and tumor-adjacent samples (from TCGA). For the first Principal Component Analysis (PCA), we were particularly interested in assessing the separation between normal tissue samples and their tumor-adjacent counterparts (Fig. 4G). The PCA results were notable: our gene signature was indeed successful in clearly demarcating these two classes of samples, affirming our earlier findings on the interconnectedness of the gene sets. Additionally, a second PCA was conducted to test the discriminatory potential of our gene signature in distinguishing both normal samples from GTEx and TCGA from BRCA (Breast Cancer) samples (Fig. 4H). This analysis was pivotal to investigate the potential clinical applicability of our findings, especially considering the pressing need for improved molecular markers in breast cancer prognosis and treatment decision-making. Once again, our gene signature demonstrated impressive performance, successfully separating normal and BRCA samples.

### Pathway and genes ontology enrichment analysis

**Figure 5:**
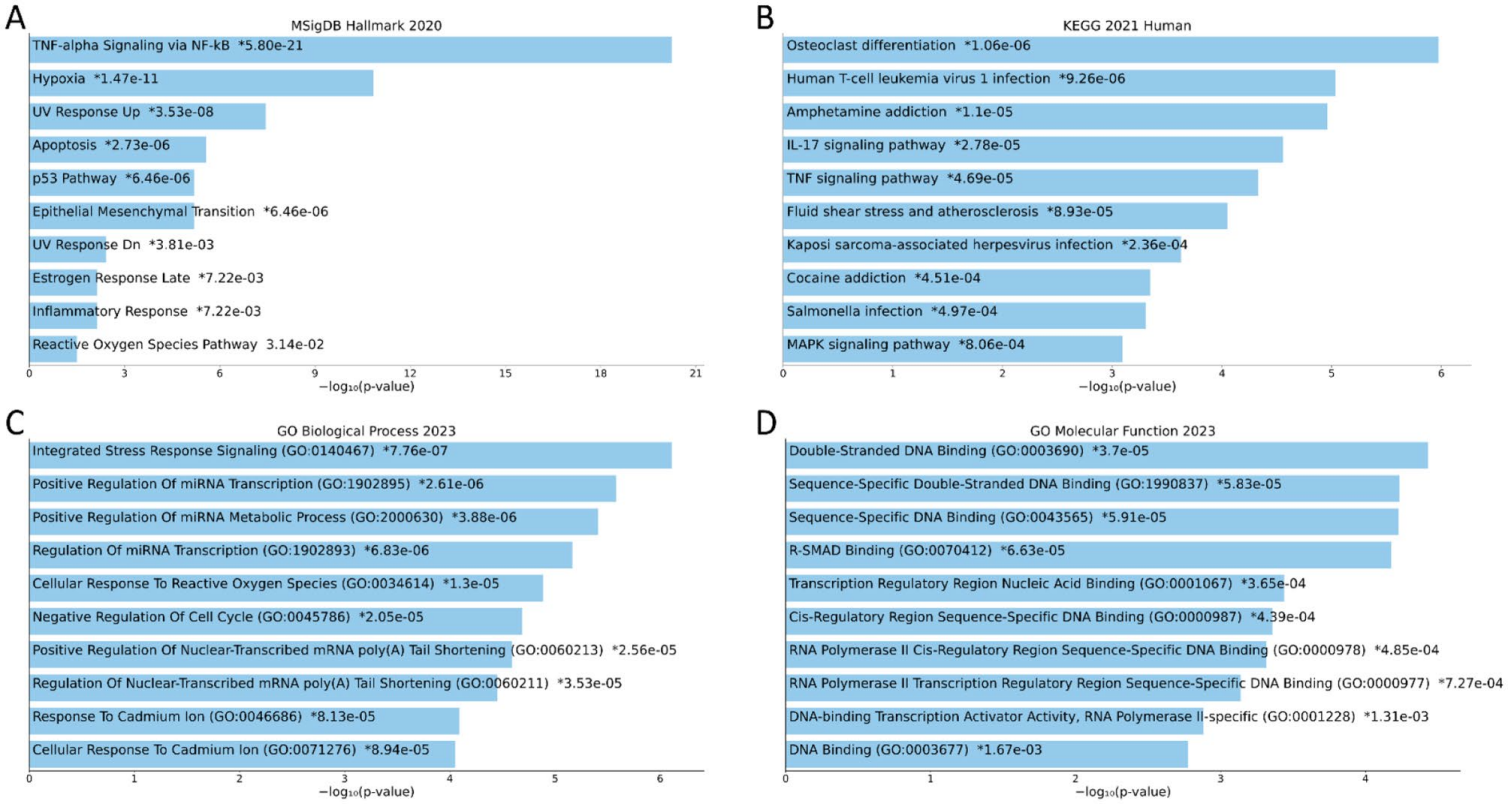
Functional enrichment analysis of the commonly regulated 13 genes. (**A**) Hallmarks enrichment analysis of the 13 genes based on the MSigDB hallmarks database. (**B**) Pathway enrichment analysis of the 13 genes based on the KEGG database. (**C**) GO Enrichment analysis of the 13 genes based on the GO biological processes database. (**D**) GO Enrichment analysis of the 13 genes based on the GO molecular function database.

To interpret the biological significance of these 13 genes, we conducted pathway enrichment analysis utilizing both the Molecular Signatures Database (MSigDB) and the Kyoto Encyclopedia of Genes and Genomes (KEGG) pathways. The most enriched MSigDB categories were TNF-alpha Signaling via NF-kB, UV Response Up, Hypoxia, Apoptosis, and the p53 Pathway (Fig. 5A). These categories suggest the downregulated genes have potential functions in regulating inflammation, responding to oxidative stress, adapting to hypoxic conditions, apoptosis, and controlling the cell cycle. The restoration of these functions through Atorvastatin treatment could be a crucial component of its proposed therapeutic efficacy in breast cancer.

The KEGG pathway analysis highlighted IL-17 Signaling and TNF Signaling pathways as being particularly enriched. These findings imply a role for the downregulated genes in inflammatory responses and cell survival regulation, both critical factors in cancer progression (Fig. 5B). The upregulation of these genes through Atorvastatin treatment might serve to modulate inflammation and cell survival, potentially contributing to its protective mechanism in breast cancer patients.

For the biological processes, the most statistically significant processes include "Positive Regulation of Nuclear-Transcribed mRNA poly(A) Tail Shortening" and "Integrated Stress Response Signaling". The former implies that Atorvastatin may modulate mRNA stability, potentially affecting the expression of various genes. The latter suggests that the drug might influence how cells respond to different stressors, which could be relevant to the harsh conditions within a tumor environment. Among other significant processes, "Regulation Of Nuclear-Transcribed mRNA poly(A) Tail Shortening" and "Negative Regulation Of Erythrocyte Differentiation" imply an involvement in mRNA dynamics and blood cell maturation respectively. "Negative Regulation of Meiotic Cell Cycle" could suggest a role in preventing the uncontrolled cell division typical of cancer cells. "Cellular Response to Granulocyte Macrophage Colony-Stimulating Factor Stimulus" and "Negative Regulation Of Monocyte Chemotaxis" might reflect potential impacts on immune cell activities, which are crucial in the tumor microenvironment (Fig. 5C).

Turning to the molecular function ontology, the most significant function is "Double-Stranded Methylated DNA Binding". This could indicate that the upregulated genes might interact with epigenetic modifications on DNA, potentially influencing gene expression patterns. Similarly, "R-SMAD Binding" suggests a role in transforming growth factor-beta (TGF-beta) signaling, a pathway often disrupted in cancer. "C–C Chemokine Binding" and "Chemokine Binding" indicate potential roles in immune cell signaling, reflecting how these genes might influence the immune response in the tumor environment. "cAMP Response Element Binding" and "Histone Acetyltransferase Binding" suggest possible roles in transcription regulation, which could help control the expression of various genes. The enrichment analysis implies that the genes upregulated by Atorvastatin might collectively modulate several key processes in breast cancer progression, including mRNA stability, stress response signaling, cell cycle control, immune system regulation, and transcription regulation (Fig. 5D). This could help explain the drug's protective effects in breast cancer patients.

### Atorvastatin reprograms the immune suppressive tumor microenvironment

**Figure 6:**
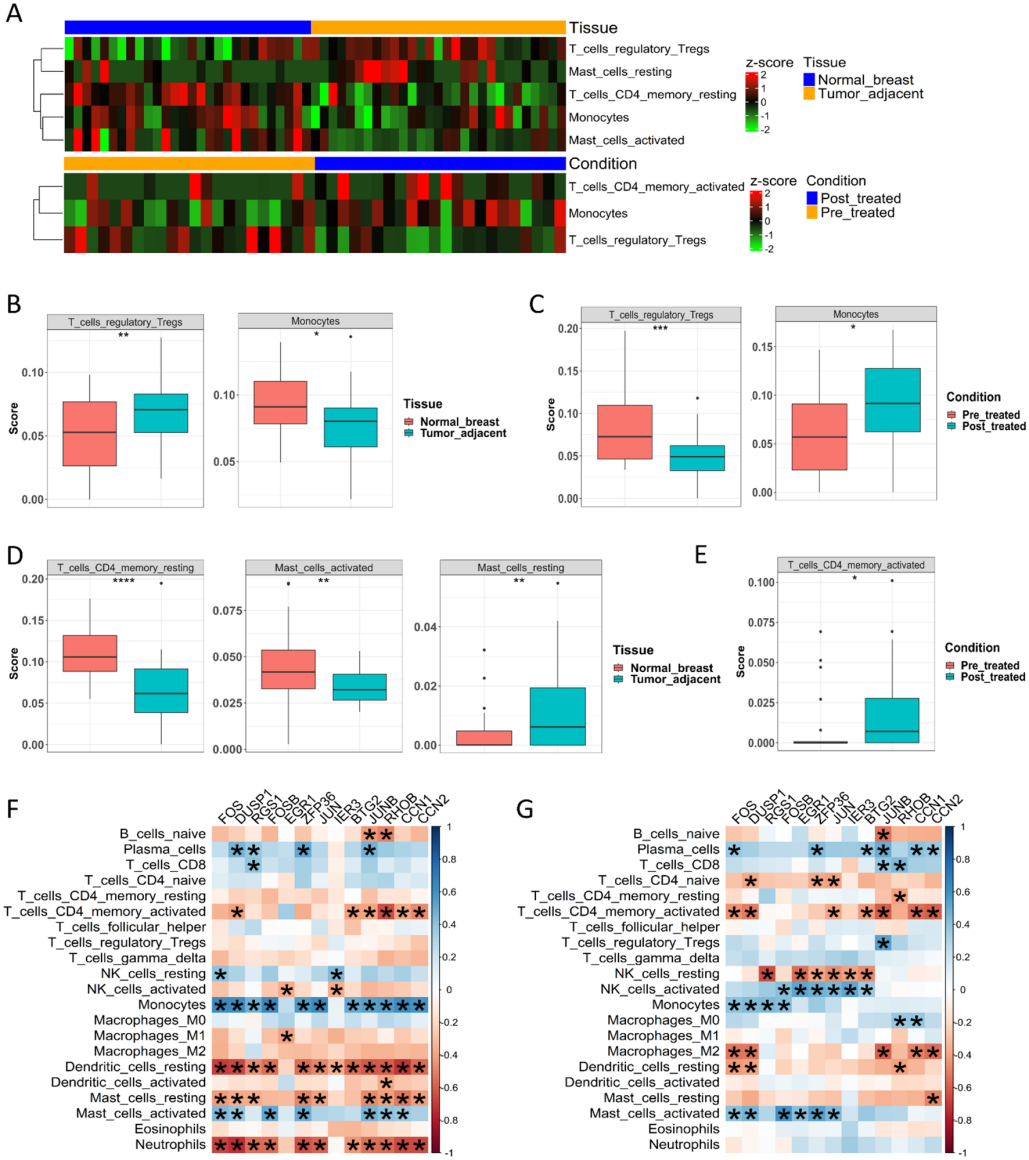
Immune deconvolution of the merged and GSE63427 datasets based on the common genes. (**A**) Heatmaps of the differentially infiltrated immune phenotypes in the merged dataset (upper) and GSE63427 dataset (lower). (**B**,**C**) The commonly differentially infiltrated immune phenotypes in merged (**B**) and GSE63427 (**C**) datasets. Atorvastatin treatment decreased T regulatory cells while increasing monocyte infiltration. (**D**) The immune phenotypes were uniquely differentially infiltrated in the merged dataset. (**E**) CD4 memory-activated T cells uniquely differentially infiltrated in the GSE63427 dataset. (**F**) Correlation between the genes expression and the infiltration of immune phenotypes tumor-adjacent samples. (**G**) Correlation between the genes expression and the infiltration of immune phenotypes normal breast samples.

Our in-depth analysis of the immune microenvironment within normal and breast cancer samples and in pre-treated and post-treated breast cancer patients using the CIBERSORTx method yielded interesting findings. We discovered a differential infiltration of five immune phenotypes in the tumor-adjacent samples when compared to normal tissue (Fig. 6A, upper), while three immune phenotypes were differentially infiltrated in the tissues of patients post-treatment compared to the pre-treatment tissues (Fig. 6A, lower). Interestingly, two immune phenotypes were found to be differentially infiltrated in both sets of tissues. One of the most notable findings was the significant increase of T regulatory cell infiltration in the tumor-adjacent samples. Conversely, a decreased level of monocyte infiltration was observed in the same samples (Fig. 6B). In a contrasting pattern, we noted decreased T regulatory cell infiltration post-treatment and increased monocyte infiltration, particularly in patients treated with Atorvastatin (Fig. 6C).

Furthermore, we identified three immune cell types that were uniquely regulated in tumor-adjacent samples. In patients treated with atorvastatin, we found one unique immune cell phenotype. Among these, T cell CD4 memory resting and mast cell activated showed a decrease in infiltration, whereas infiltration of immune suppressive resting mast cells increased (Fig. 6D). Importantly, Atorvastatin treatment was associated with an increased infiltration of CD4 T cell memory activated (Fig. 6E). As part of a deeper investigation, we analyzed the correlation between 13 specific genes and the infiltration of these immune phenotypes, focusing on discerning the differential patterns in normal breast samples and tumor-adjacent samples. Our correlation analysis revealed distinct patterns that were unique to the tumor-adjacent tissues.

In the tumor-adjacent tissue, most of the genes were negatively correlated with the infiltration of neutrophils, resting mast cells, and resting dendritic cells. However, these genes displayed a positive correlation with monocyte infiltration (Fig. 6F). Approximately half of the genes were positively correlated with activated mast cells. Interestingly, this pattern was found to be exclusive to the tumor-adjacent tissues (Fig. 6F,G). About half of the genes showed a positive correlation with the infiltration of activated natural killer cells and activated mast cells.

### The gene signature downregulation associates with tumor aggressiveness and TP35 mutations

We explored the expression pattern of 13 genes in breast cancer samples obtained from The TCGA using cBioPortal. Our analysis revealed that the expression of these genes was significantly altered in 88% (959) of the samples.

**Figure 7:**
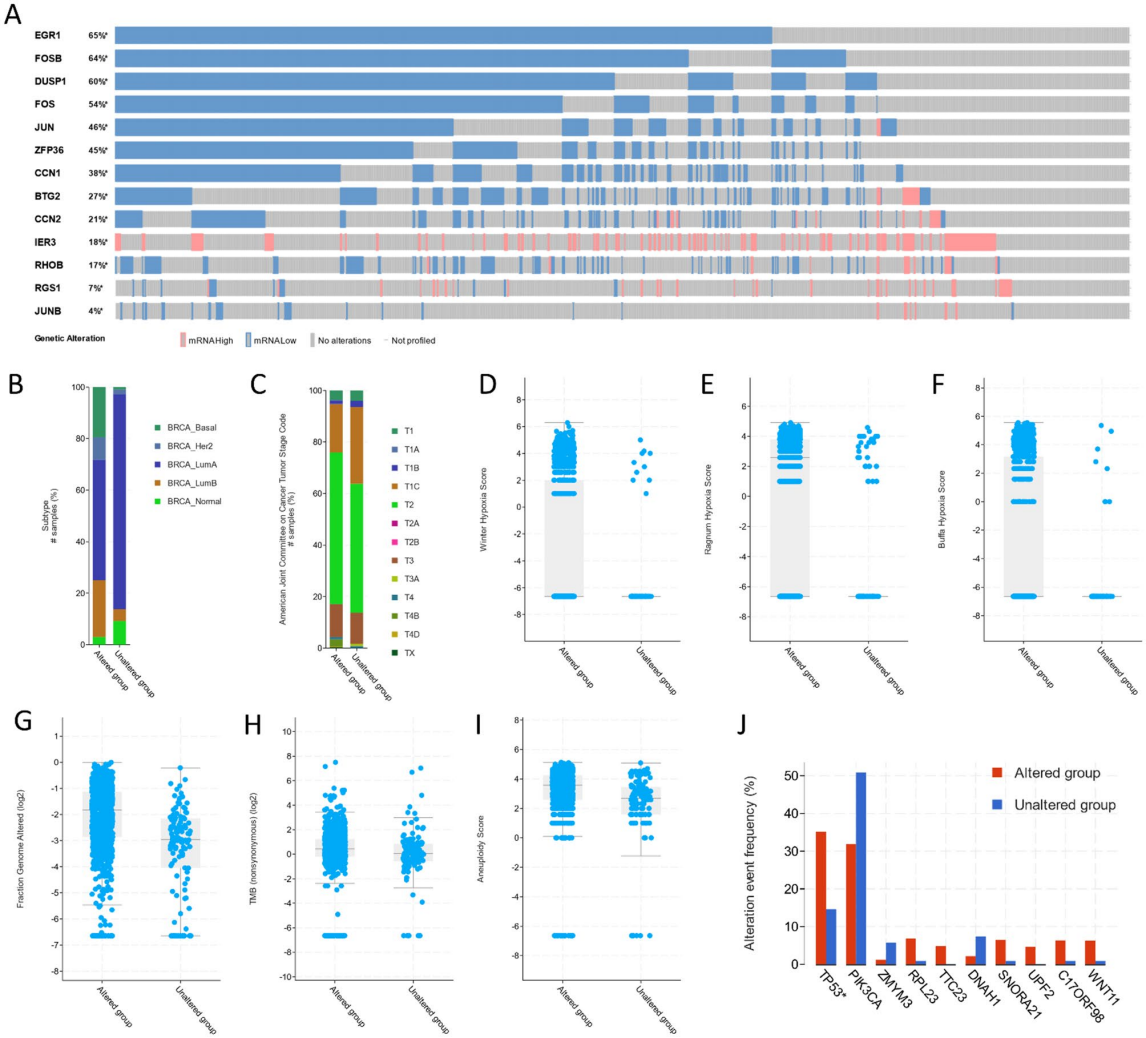
Exploring the 13 genes in TCGA-BRCA cohort via the cBioportal database. (**A**) Gene expression alterations of the 13 genes in BRCA samples (z = ± 2). Genes ranked according to the percentage of altered samples. (**B**) The correlation between genes expression alterations and breast cancer subtypes. The altered group has a higher percentage of aggressive phenotypes (BRCA-Basal, BRCA-HER2, and BRCA-LumB) patients while the opposite in less aggressive phenotypes (BRCA-Normal and BRCA-LumA). (**C**) The correlation between gene expression alterations and the breast cancer stages. The altered group has a higher percentage of more advanced stage samples, while exclusively has samples in the most advanced stages (T4B-TX). (**D**–**F**) The relation between gene expression alterations and the hypoxia score. The altered group has higher winter, ragnum, and buffa scores. (**G**) The relation between gene expression alterations and fraction of genome altered. The altered group has greater fraction of the genome altered. (**H**) The relation between gene expression alterations and the tumor mutation burden (TMB). The altered group has higher TBM. (**I**) The relation between gene expression alterations and aneuploidy. The altered group has higher aneuploidy. (**J**) The association between gene expression alterations and the enriched mutations. TP53 is mutations are significantly enriched in the altered group.

Firstly, we observed that the expression levels of the 13 genes exhibited significant downregulation in the majority of the samples, except for IER3 and RGS1, which deviated from this pattern (Fig. 7A). Next, we investigated the relationship between the expression alterations and breast cancer subtypes. We found that the altered group had a higher percentage of aggressive phenotypes, including basal, HER2, and luminal B subtypes, while the unaltered group was enriched for luminal A and normal-like phenotypes (Fig. 7B)This observation suggests that the expression alterations of these genes may be associated with the development and progression of more aggressive subtypes of breast cancer.

Furthermore, we examined the association between the expression alterations and cancer stages. Strikingly, the altered group contained samples with more advanced stages (T4A-T4D) of breast cancer, while the unaltered group did not include any samples in such advanced stages (Fig. 7C). This finding suggests that the dysregulation of these genes may be linked to tumor progression and is potentially indicative of their involvement in the later stages of breast cancer. Moreover, we evaluated the relationship between expression alterations and hypoxia scores. The altered group exhibited higher hypoxia scores compared to the unaltered group (Fig. 7D–F). Hypoxia, which represents low oxygen levels, is known to influence cancer aggressiveness and is linked to poor prognosis. Thus, the higher hypoxia scores in the altered group further support the potential role of these genes in driving tumor aggressiveness.

**Figure 8:**
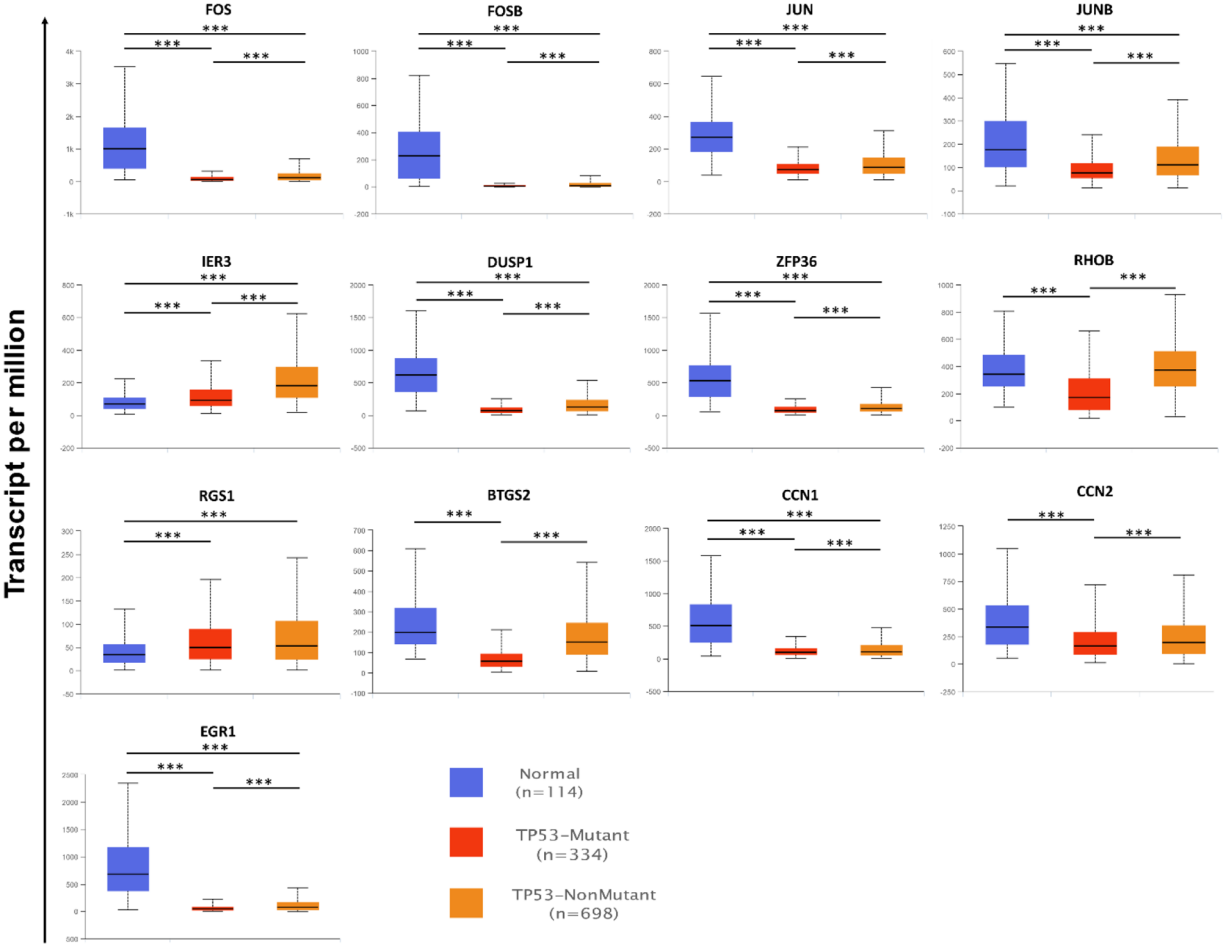
Detailed analysis of the relation between gene expression and TP53 mutations. All the genes were significantly lower in the TP53 mutant group except RGS1.

We also assessed genomic alterations in relation to the expression alterations. The altered group showed a higher fraction of the genome altered (Fig. 7G), increased tumor mutational burden (Fig. 7H), and higher aneuploidy scores (Fig. 7I). These genomic instability indicators are commonly associated with aggressive and advanced cancers, providing further evidence for the role of these genes in cancer progression. Finally, we investigated the association between expression alterations and TP53 mutation status. TP53 is a crucial tumor suppressor gene frequently mutated in cancer. Our analysis demonstrated that the group with TP53 mutation had significantly lower expression levels of the 13 genes compared to the unmutated group (Fig. 7J). This finding suggests a potential link between TP53 mutation and the downregulation of these genes, highlighting the interplay between TP53 and the dysregulation of these genes in breast cancer. Further analysis using UALCAN database shoed that genes’ expression values were in the TP53 mutant samples (Fig. 8).

### Collective gene expression alterations of the 13 genes correlates with activated cell cycle and altered ECM

**Figure 9:**
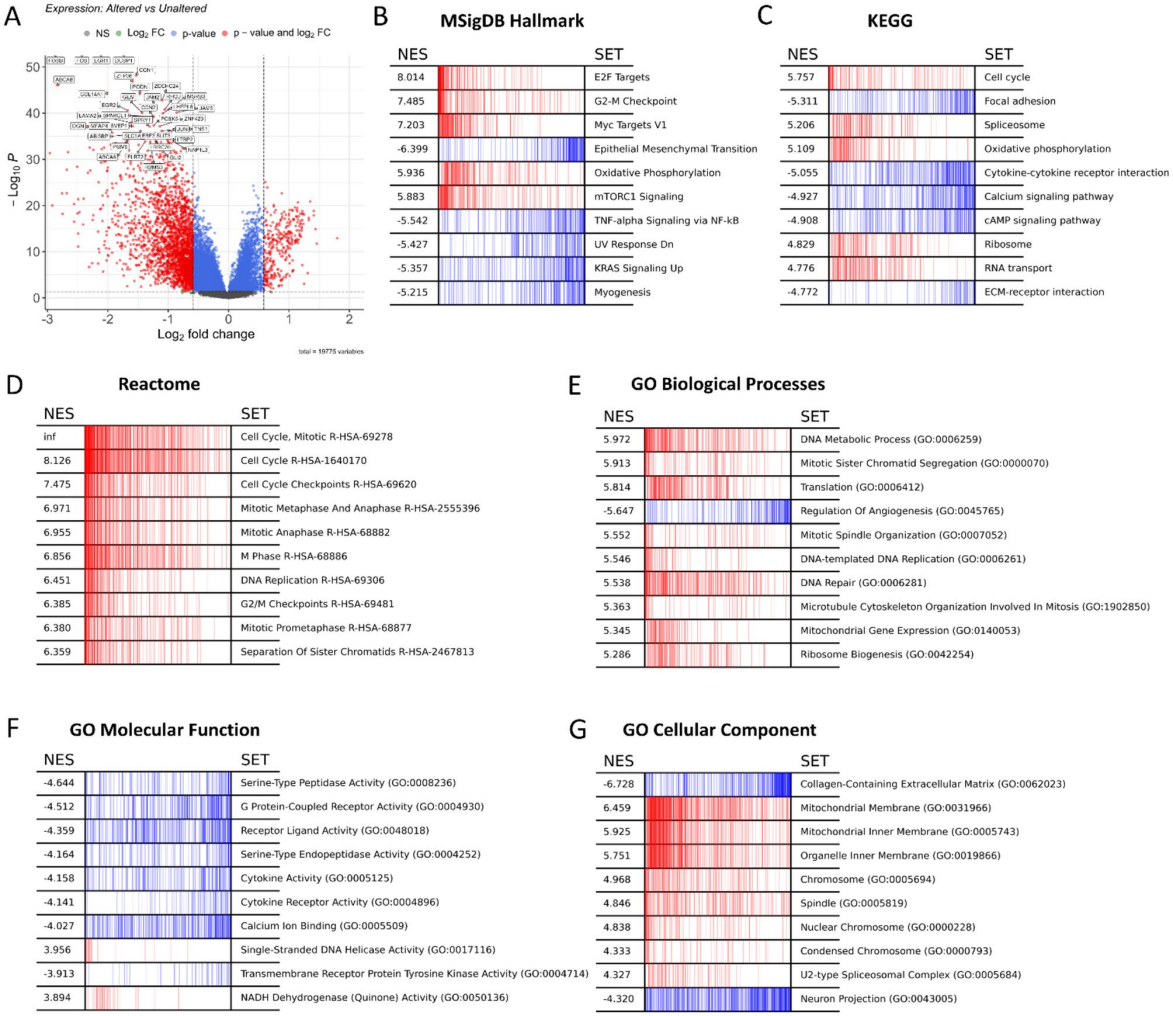
GSEA of the DEGs between altered and unaltered groups. (**A**) Volcano plot of the DEGs due to the gene expression alterations of the 13 genes. (**B**) Hallmarks GSEA of the DEGs due to the 13 gene alterations based on the MSigDB hallmarks database. (**C**) Pathway GSEA of the DEGs due to the 13 gene alterations based on the KEGG database. (**D**) Pathway GSEA of the DEGs due to the 13 gene alterations based on the Reactome database. (**E**) GO GSEA of the DEGs due to the 13 gene alterations based on GO biological processes databse. (**F**) GO GSEA of the DEGs due to the 13 gene alterations based GO moleculsar function database. (**G**) GO GSEA of the DEGs due to the 13 gene alterations based on the GO cellular component database. Red indicates positive enrichment score while blue indicates negative enrichment score.

Our findings were further validated by leveraging the cBioPortal platform to explore the expression alterations of the 13 identified genes in the TCGA-BRCA and the transcriptional impact of these alterations. Comparing the altered and unaltered groups, we screened the DEG s due to the 13 gene signature alterations To further understand their impact, we performed GSEA on the identified DEGs. The results of our analysis suggest a significant association between the transcriptional alterations of 13 specific genes and the therapeutic action of Atorvastatin against breast cancer. The expression alteration displayed a substantial impact on transcriptional activities as visualized in our volcano plot (Fig. 9A). When examined further for biological relevance, these genes, via MSigDB Hallmark GSEA, showed enrichment in pathways primarily related to cell cycle activation such as E2F targets, G2-M checkpoint, Myc targets, oxidative phosphorylation, and mTORC1 signaling (Fig. 9B).

Interestingly, the DEGs between the altered and unaltered groups showed a reduced representation in pathways related to epithelial-mesenchymal transition. This observation could hint at the protective and potentially toxic effects mediated through the upregulation of the 13 genes, leading to the suppression of cell cycle-related processes. It also provides a potential explanation for the association observed between long-term Atorvastatin usage and an increased rate of metastasis. Our KEGG analysis and Reactome analysis further emphasized the enrichment of genes in cell cycle-related pathways and oxidative phosphorylation, while showing a decreased representation in focal adhesion and extracellular matrix-receptor interaction (Fig. 9C,D). GO biological processes enrichment analysis also revealed similar trends with the enrichment of genes in cell cycle-related processes, DNA metabolic processes, and mitotic spindle organization, while displaying depletion in angiogenesis (Fig. 9E). From a molecular function perspective, the genes were enriched in single-stranded DNA helicase activity and were depleted in signaling-related molecular functions, such as G-protein-coupled receptor activity and cytokine activity (Fig. 9F). These genes were majorly localized in mitochondrial and nuclear components, while showing a reduced presence in ECM components (Fig. 9G).

### The 13 gene panel lower expression predicts worse prognosis

**Figure 10:**
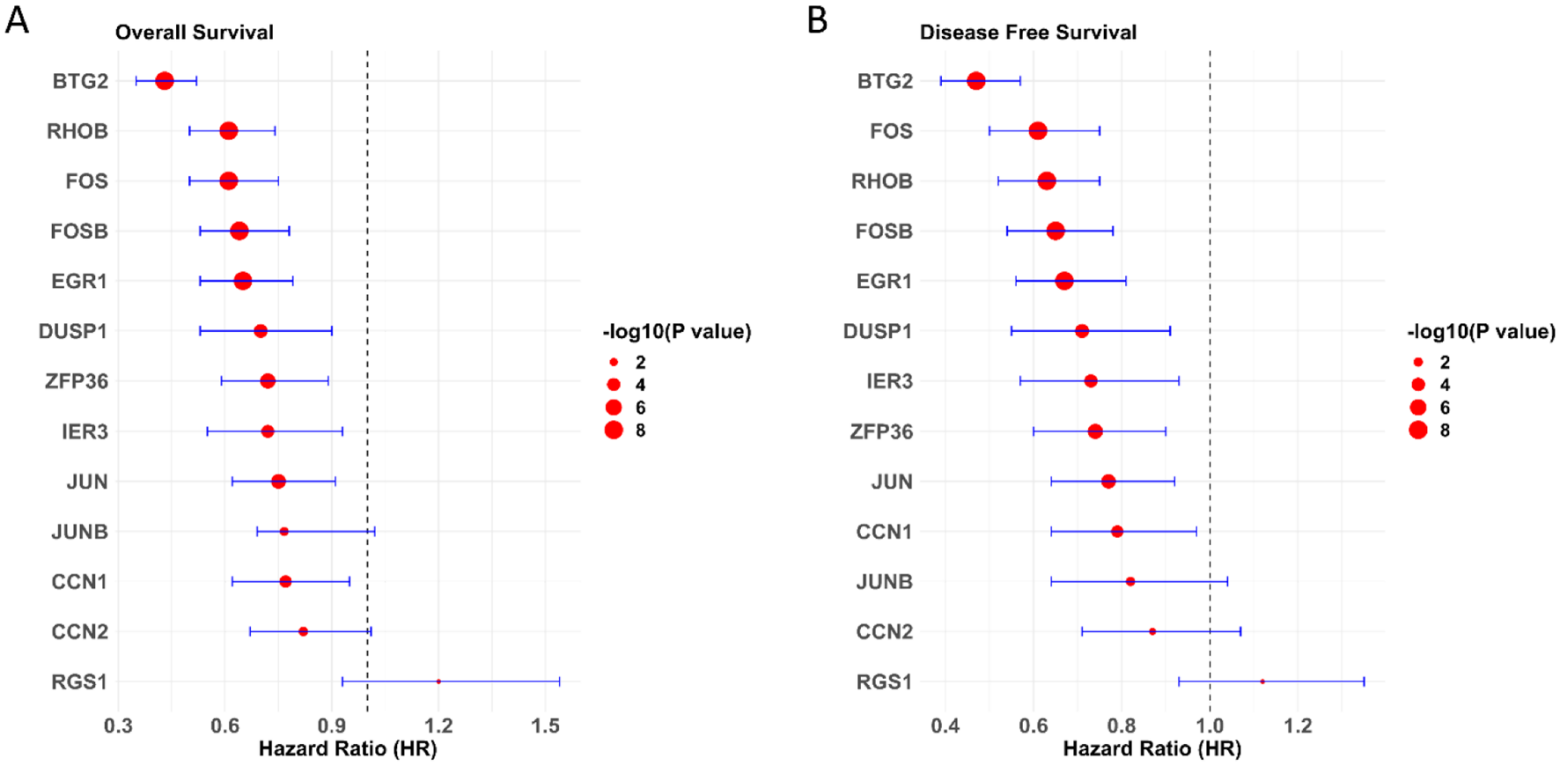
Survival analysis of the 13 genes based on the RNA seq samples in bcgenexminer database. (**A**) Overall survival analysis of the 13 genes. Genes were mostly predicting better prognosis. (**B**) Disease free survival of the 13 genes. Genes were mostly predicting better prognosis.

We conducted survival analysis for a panel of the 13 genes using the exhaustive module in bcgenexminer database. This investigation evaluated the prognostic significance of these genes by conducting both OS and DFS analyses. Most of the genes (12 out of 13) demonstrated a hazard ratio (HR) lower than one in both OS and DFS analyses (Fig. 10). A HR less than one typically suggests a decreased risk of death or disease recurrence, implying that high expression of these genes could potentially serve as a favorable prognostic factor in breast cancer. Furthermore, Atorvastatin could potentially enhance patient survival by reducing the risk of death or disease recurrence by upregulating those genes.

## Discussion

Batch effects, caused by non-biological variations across datasets, sometimes invalidate multi-dataset analysis. The ComBat function of the R sva package was used to correct such effects in this investigation. The absence of dataset separation post-ComBat application highlights the merged datasets' consistency and homogeneity. 116 DEGs between normal and tumor-adjacent tissues offer a basis for comprehending breast carcinogenesis' molecular subtleties. The observed up- and downregulation patterns suggest processes that may cause normal breast tissues to become tumorigenic. These results underline the differences between the two tissue types and enable focused treatment research using DEGs. While the preliminary assessment of the GSE63427 data did not indicate a significant impact of atorvastatin, the discovery of 105 DEGs post-treatment gives important insights into the drug's molecular actions. The heatmap clustering shows atorvastatin's slight but noticeable effect. PCA findings indicated low differentiation between pre- and post-treated samples, which may indicate the drug's subtle transcriptional regulation rather than a transcriptome reprogramming.

The possible breast cancer protection of Atorvastatin was our main focus. Our notion is supported by the discovery of 13 genes shared by atorvastatin-regulated and tumor-adjacent dysregulated genes. The hypergeometric test's substantial *p* value (1.57e-13) proves that the 13 often regulated genes are biologically important. This, together with the highly linked PPI network (enrichment *p* value < 1.0e-16), supports coordinated gene activity. Their substantial interconnectedness suggests shared or cooperative functioning, stressing the need of full gene set analysis for downstream research. The PCA findings from GEPIA2 are impressive, distinguishing normal tissue samples from tumor-adjacent ones and GTEx and TCGA from BRCA samples. This gene signature's outstanding performance has major therapeutic implications, opening new possibilities for breast cancer prognostic and therapy molecular indicators.

The enriched MSigDB categories and KEGG pathways revealed the 13-gene signature's several activities, including inflammation regulation, oxidative stress response, hypoxia adaption, apoptosis, and cell cycle control. These data support atorvastatin's breast cancer treatment effectiveness. Atorvastatin modulates mRNA stability, stress response signaling, cell cycle control, and immune system regulation, according to statistically significant biological processes. Molecular function ontology highlights the gene signature's importance in DNA binding, TGF-beta signaling, immune cell signaling, and transcription control, revealing atorvastatin's protective properties.

Our work highlights the dynamic immunological microenvironment-atorvastatin interaction in breast cancer. The increased T regulatory cell infiltration in tumor-adjacent tissues implies an immune suppressive milieu that may encourage carcinogenesis. Remarkably, post-atorvastatin therapy showed reversed patterns, suggesting the medicine may change this suppressive milieu to favor anti-tumor immunity. Atorvastatin's unique immune cell regulation, specifically CD4 T cell memory stimulated infiltration, enhances adaptive immunity. Additionally, the unique connection patterns between the 13 genes and immune cells in tumor-adjacent tissues demonstrate the complex interaction between genetic determinants and the immunological landscape. These data suggest that Atorvastatin may treat breast cancer and that its immunomodulatory mechanisms deserve additional study.

The gene expression patterns of the 13 genes explored in the TCGA-BRCA samples suggest their possible role in carcinogenesis and progression. The widespread downregulation of these genes, except for IER3 and RGS1, suggests additional study. Their connection with aggressive breast cancer phenotypes, such as basal, HER2, and luminal B subtypes, implies that dysregulation may contribute to cancer development. Since the changed expression group mostly includes advanced breast cancer stages, gene dysregulation is linked to disease severity. The changed group's higher hypoxia scores support the link between altered gene expression and cancer aggressiveness, since hypoxia promotes tumor malignancy^46^. The genomic instability, which includes greater tumor mutational load, higher aneuploidy scores, and widespread genome changes, supports these genes' potential as markers or facilitators of aggressive tumor activity. The link between TP53 mutant status and reduced 13 gene expression is especially interesting. Given TP53's importance in genomic integrity and its frequent mutation in malignancies^47,48^, our results suggest a molecular link between TP53 mutation and gene dysregulation. These findings emphasize the need to study the molecular mechanisms governing these genes.

We can better comprehend breast cancer's cellular dynamics by studying gene expression patterns and pharmacological actions like atorvastatin. Our work, using TCGA-BRCA data from cBioPortal, showed that the 13-gene signature modification had a widespread transcriptional effect on breast cancer cells. Our GSEA revealed the enrichment of cell cycle activation pathways such E2F, G2-M checkpoint, and Myc. These pathways govern cell proliferation and are often disrupted in cancer^49^. The importance of oxidative phosphorylation and mTORC1 signaling highlights metabolic changes that may be promoting cancer cell aggression. An interesting counterbalance is the lower prevalence in epithelial-mesenchymal transition (EMT) pathways. EMT is important for cancer metastasis, therefore repressing it may seem beneficial^50^. However, atorvastatin's long-term use and correlation with increased metastasis suggest that this reduced EMT representation may be a nuanced indicator of an altered tumor microenvironment that adapts to therapeutic interventions, bypassing traditional EMT routes or using alternative metastatic pathways.

The KEGG and Reactome analysis reinforced the aforementioned trends, spotlighting the dominance of cell cycle and oxidative phosphorylation pathways. The decline in focal adhesion and extracellular matrix-receptor interactions emphasizes altered cell-environment communications, which could have implications for cell mobility, tissue invasion, and drug resistance^51^. From a GO perspective, the enrichment trends underscored a cancer cell's enhanced emphasis on DNA replication, mitosis, and cellular energetics, all crucial for rapid proliferation. The diminished representation in angiogenesis, however, suggests potential vulnerabilities that could be therapeutically exploited. It's noteworthy that these genes were mainly found in mitochondrial and nuclear locales, which are the hubs of energy production and genetic regulation, respectively, emphasizing their potential role in shaping the cancer cell's phenotype.

Survival analysis plays a fundamental role in the field of oncological research, offering crucial insights into the prognostic significance of particular molecular markers. The results obtained provide solid evidence for the prognostic importance of the 13-gene panel in breast cancer. Twelve genetic variables showed HRs smaller than one in both OS and DFS. This insight holds significant value. A HR below one often signifies a diminished probability of unfavorable occurrences, such as mortality, linked to elevated gene expression levels. Hence, within the realm of breast cancer, heightened levels of gene expression may be regarded as biomarkers indicative of a positive prognosis.

The potential beneficial impact of these genes is further heightened by the observed association with atorvastatin. Drawing a link between the upregulation of these genes and Atorvastatin introduces a novel perspective on the therapeutic value of this drug. While Atorvastatin is primarily known for its lipid-lowering effects, emerging evidence suggests its potential anti-tumorigenic properties in various cancers^52^. The evidence presented in this study provides support for the hypothesis that Atorvastatin may improve patient survival in breast cancer through the upregulation of genes that have the ability to reduce death rates and decrease the likelihood of disease recurrence.

In conclusion, the collective evidence from our survival analysis underscores the potential prognostic value of the 13-gene panel in breast cancer. While the mechanistic link between Atorvastatin and the expression of these genes warrants further exploration, the preliminary findings establish a promising foundation for future therapeutic interventions and prognostic assessments.

### Supplementary Information

Supplementary Information 1.

Supplementary Information 2.

Supplementary Information 3.

Supplementary Information 4.

Supplementary Tables.

## Data Availability

The datasets downloaded and analysed during the current study are available in the Gene Expression Omnibus (GEO) repository, and can be direclt accessed using https://www.ncbi.nlm.nih.gov/geo/query/acc.cgi?acc=GSE9574, https://www.ncbi.nlm.nih.gov/geo/query/acc.cgi?acc=GSE20437, and https://www.ncbi.nlm.nih.gov/geo/query/acc.cgi?acc=GSE63427.

## Abbreviations

CSM: Consensus similarity-based model
DEGs: Differentially expressed genes
DFS: Disease-free survival
FDR: False discovery rate
GEO: Gene Expression Omnibus
GI_50%_: Growth inhibition 50%
GO: Gene ontology
GSEA: Gene set enrichment analysis
HMG-CoA: 3-Hydroxy-3-methylglutaryl coenzyme A
HR: Hazard ratio
JNK: C-Jun N-terminal kinase
KEGG: Kyoto Encyclopedia of Genes and Genomes
LINCS: Library of Integrated Network-Based Cellular Signatures
MCC: Maximal clique centrality
MDS: Molecular dynamics simulation
MSigDB Hallmarks: Molecular Signature Data Base Hallmarks
OS: Overall survival
PPI: Protein–protein interaction
QSAR: Quantitative structure–activity relationship
RG: Radius of Gyration
RMSD: Root mean square deviation
RMSF: Root mean square fluctuation
ROC: Receiver operating characteristic
SASA: Solvent accessible surface area
STRING: Search Tool for the Retrieval of Interacting Genes/Proteins
TFEA: Transcription factor enrichment analysis
VEGF: Vascular endothelial growth factor
