# Supplementary material for: Atorvastatin lowers breast cancer risk by reversing an early tumorigenic signature
Source: Sci Rep. 2024 Aug 1;14:17803. doi: 10.1038/s41598-024-67706-2 (PMC11294600; doi:10.1038/s41598-024-67706-2)
Supplement: Supplementary file 5 — Supplementary Tables. [file 41598_2024_67706_MOESM5_ESM.docx]

| Study | array | sample | 1* | 2* | 3* | 4* | 5* | 6* | Study | sample | 1* | 2* | 3* | 4* | 5* | 6* |
| --- | --- | --- | --- | --- | --- | --- | --- | --- | --- | --- | --- | --- | --- | --- | --- | --- |
| **GSE9574** | 1 | GSM241999 |  |  |  |  |  |  | **GSE20437** | GSM512539 |  |  |  |  |  |  |
|  | 2 | GSM242000 |  |  |  |  |  |  |  | GSM512540 |  |  |  |  | x |  |
|  | 3 | GSM242001 |  |  |  |  |  |  |  | GSM512541 |  |  |  |  |  |  |
|  | 4 | GSM242002 |  |  |  |  |  |  |  | **GSM512542** | x | x |  |  |  |  |
|  | 5 | **GSM242003** | x |  |  |  | x |  |  | GSM512543 |  |  |  |  | x |  |
|  | 6 | GSM242004 |  |  |  |  |  |  |  | GSM512544 |  |  |  |  |  |  |
|  | 7 | GSM242005 |  |  |  |  |  | x |  | GSM512545 | x |  |  |  |  |  |
|  | 8 | GSM242006 |  |  |  |  |  |  |  | GSM512546 |  |  |  |  |  |  |
|  | 9 | GSM242007 |  |  |  |  |  |  |  | **GSM512547** | x |  |  |  | x |  |
|  | 10 | GSM242008 |  |  |  |  |  |  |  | **GSM512548** | x | x |  |  |  |  |
|  | 11 | GSM242009 |  |  |  |  |  |  |  | GSM512549 |  |  |  |  |  |  |
|  | 12 | GSM242010 |  |  |  |  |  |  |  | GSM512550 |  |  |  |  | x |  |
|  | 13 | GSM242011 | x |  |  |  |  |  |  | GSM512551 |  |  |  |  |  |  |
|  | 14 | GSM242012 |  |  |  |  |  |  |  | GSM512552 |  |  |  |  |  |  |
|  | 15 | **GSM242013** | x |  |  |  | x |  |  | GSM512553 |  |  |  |  |  |  |
|  | 16 | GSM242014 |  |  |  |  |  |  |  | GSM512554 |  |  |  |  | x |  |
|  | 17 | GSM242015 |  |  |  |  | x |  |  | GSM512555 |  |  |  |  |  |  |
|  | 18 | GSM242016 |  |  | x |  |  |  |  | GSM512556 |  |  |  |  |  |  |
|  | 19 | GSM242017 |  |  |  |  |  |  |  | GSM512557 |  |  |  |  |  |  |
|  | 20 | GSM242018 |  |  |  |  | x |  |  | GSM512558 |  |  |  |  | x |  |
|  | 21 | GSM242019 |  |  | x |  |  |  |  | GSM512559 |  |  |  |  |  |  |
|  | 22 | **GSM242020** | x |  |  |  | x |  |  | GSM512560 |  |  |  |  | x |  |
|  | 23 | **GSM242021** | x |  |  |  | x |  |  | GSM512561 |  |  | x |  |  |  |
|  | 24 | GSM242022 |  |  |  |  | x |  |  | GSM512562 |  |  |  |  |  |  |
|  | 25 | GSM242023 |  |  |  |  | x |  |  | GSM512563 |  |  |  |  |  |  |
|  | 26 | GSM242024 |  |  |  |  | x |  |  | GSM512564 |  |  |  | x |  |  |
|  | 27 | GSM242025 |  |  |  |  | x |  |  | GSM512565 |  |  |  |  | x |  |
|  | 28 | GSM242026 |  |  |  |  |  |  |  | GSM512566 |  |  |  |  |  |  |
|  | 29 | GSM242027 |  |  |  |  | x |  |  | GSM512567 |  |  |  |  |  |  |
|  | 30 |  |  |  |  |  |  |  |  | GSM512568 |  |  |  |  | x |  |
|  |  |  |  |  |  |  |  |  |  | GSM512569 |  |  |  |  |  |  |
|  |  |  |  |  |  |  |  |  |  | GSM512570 |  |  |  |  |  | x |
|  |  |  |  |  |  |  |  |  |  | GSM512571 |  |  |  |  |  |  |
|  |  |  |  |  |  |  |  |  |  | GSM512572 |  |  |  |  |  |  |
|  |  |  |  |  |  |  |  |  |  | GSM512573 |  |  |  |  |  |  |
|  |  |  |  |  |  |  |  |  |  | GSM512574 |  |  |  |  |  |  |

Table S1 First round sample quality metrics

1. outlier detection by Distances between arrays
2. outlier detection by Boxplots
3. outlier detection by Relative Log Expression (RLE)
4. outlier detection by Normalized Unscaled Standard Error (NUSE)
5. outlier detection by MA plots
6. outlier detection by Spatial distribution of M

Table S2. Second round sample quality metrics

| Study | array | sample | *1 | *2 | *3 | Study | array | sample | *1 | *2 | *3 |
| --- | --- | --- | --- | --- | --- | --- | --- | --- | --- | --- | --- |
| **GSE9547** | 1 | GSM241999 |  |  |  | **GSE20437** | 1 | GSM512539 | |  |  |
|  | 2 | GSM242000 |  |  |  |  | 2 | GSM512540 | |  |  |
|  | 3 | GSM242001 |  |  |  |  | 3 | GSM512541 | |  |  |
|  | 4 | GSM242002 |  |  |  |  | 4 | GSM512543 | |  |  |
|  | 5 | GSM242004 |  |  |  |  | 5 | GSM512544 | |  |  |
|  | 6 | GSM242005 |  |  |  |  | 6 | GSM512545 | |  |  |
|  | 7 | GSM242006 |  |  |  |  | 7 | GSM512546 | |  |  |
|  | 8 | GSM242007 |  |  |  |  | 8 | GSM512549 | |  |  |
|  | 9 | GSM242008 |  |  |  |  | 9 | GSM512550 | |  |  |
|  | 10 | GSM242009 |  |  |  |  | 10 | GSM512551 | |  |  |
|  | 11 | GSM242010 |  |  |  |  | 11 | GSM512552 | |  |  |
|  | 12 | GSM242011 |  |  |  |  | 12 | GSM512553 | |  |  |
|  | 13 | GSM242012 |  |  |  |  | 13 | GSM512554 | |  |  |
|  | 14 | GSM242014 |  |  |  |  | 14 | GSM512555 | |  |  |
|  | 15 | GSM242015 |  |  |  |  | 15 | GSM512556 | |  |  |
|  | 16 | **GSM242016** | x |  |  |  | 16 | GSM512557 | |  |  |
|  | 17 | GSM242017 |  |  |  |  | 17 | GSM512558 | |  |  |
|  | 18 | GSM242018 |  |  |  |  | 18 | GSM512559 | |  |  |
|  | 19 | GSM242019 |  |  |  |  | 19 | GSM512560 | |  |  |
|  | 20 | GSM242022 |  |  |  |  | 20 | GSM512561 | |  |  |
|  | 21 | GSM242023 |  |  |  |  | 21 | GSM512562 | |  |  |
|  | 22 | GSM242024 |  |  |  |  | 22 | GSM512563 | |  |  |
|  | 23 | GSM242025 |  |  |  |  | 23 | GSM512564 | |  |  |
|  | 24 | GSM242026 |  |  |  |  | 24 | GSM512565 | |  |  |
|  | 25 | GSM242027 |  |  |  |  | 25 | GSM512566 | |  |  |
|  |  |  |  |  |  |  | 26 | GSM512567 | |  |  |
|  |  |  |  |  |  |  | 27 | GSM512568 | |  |  |
|  |  |  |  |  |  |  | 28 | GSM512569 | |  |  |
|  |  |  |  |  |  |  | 29 | GSM512570 | |  |  |
|  |  |  |  |  |  |  | 30 | GSM512571 | |  |  |
|  |  |  |  |  |  |  | 31 | GSM512572 | |  |  |
|  |  |  |  |  |  |  | 32 | GSM512573 | |  |  |
|  |  |  |  |  |  |  | 33 | GSM512574 | |  |  |

1. outlier detection by [Distances between arrays](file:///F:\Breast%20cancer%20diagnostic%20signature%20generation\Final%20analysis\expData_GSE20437_1st_QC_pass\index.html#hm)
2. outlier detection by [Boxplots](file:///F:\Breast%20cancer%20diagnostic%20signature%20generation\Final%20analysis\expData_GSE20437_1st_QC_pass\index.html#box)
3. outlier detection by [MA plots](file:///F:\Breast%20cancer%20diagnostic%20signature%20generation\Final%20analysis\expData_GSE20437_1st_QC_pass\index.html#ma)
